# Defocusing-Controlled Morphology and Electrochemical Performance of Laser-Induced Graphene Electrodes for Microsupercapacitors

**DOI:** 10.3390/polym18192377

**Published:** 2026-09-29

**Authors:** Seon Jong Yu, Da Hwan Yu, Mun Jeong Choi, Inhwan Lee, Jungho Cho, Hyoryung Nam, Geon Hwee Kim

**Affiliations:** 1 School of Mechanical Engineering, Chungbuk National University (CBNU), 1, Chungdae-ro, Seowon-gu, Cheongju-si 28644, Republic of Korea; sunzong72@gmail.com (S.J.Y.); dugang@chungbuk.ac.kr (D.H.Y.); anxanx@chungbuk.ac.kr (I.L.); junghocho@chungbuk.ac.kr (J.C.); 2 Department of Mechanical Engineering, Chungbuk National University (CBNU), 1, Chungdae-ro, Seowon-gu, Cheongju-si 28644, Republic of Korea; cmjanswjd@naver.com; 3 Department of Biomedical Engineering, Daegu Catholic University School of Medicine, 33 Duryugongwon-ro 17-gil, Nam-gu, Daegu 42472, Republic of Korea

**Keywords:** laser-induced graphene, polyimide, polymer electrolyte, defocusing, carbonization, microsupercapacitor, energy storage

## Abstract

Laser-induced graphene (LIG) is a promising porous carbon electrode for flexible microsupercapacitors (MSCs), and defocusing is an established means of tuning its morphology. Here, a defocusing series was evaluated as a complete process-to-device chain with the CO_2_ laser power and scan speed fixed at 5.0 W and 46 mm s^−1^. A polyimide substrate was positioned 0–3 mm below the focal plane to produce laser-induced graphene fibers (LIGFs), LIG-1, LIG-2, and LIG-3. Increasing the defocusing distance lowered the peak fluence per pulse from 16.7 to 2.1 J cm^−2^, transforming a fibrous network into porous LIG, an open-cell-like network, and a film whose smooth, oxygen-rich surface skin covered a porous interior. LIG-2 showed the lowest sheet resistance (15.3 Ω sq^−1^), I_D_/I_G_ ratio (0.29), and equivalent series resistance (78 Ω), delivering 2.88 mF cm^−2^ at 0.05 mA cm^−2^ and 97.4% retention after 2000 cycles. LIGF showed the lowest contact angle (12.1°), retained 57.3% of its capacitance at 0.25 mA cm^−2^, and surpassed LIG-2 at ≥0.10 mA cm^−2^. Both electrodes retained their cyclic voltammetry (CV) response under a single 180° bend. Thus, substrate-height adjustment tunes LIG morphology and rate-dependent MSC performance without changing laser power or scan speed.

## 1. Introduction

The rapid development of wearable electronics and the Internet of Things (IoT) has increased the demand for compact, lightweight, and flexible energy-storage devices. Lithium-ion batteries provide a high energy density, but their relatively large volume, the safety issues associated with liquid electrolytes, and limited resource availability make them difficult to integrate directly into small-scale electronics. Microsupercapacitors (MSCs), which offer rapid charge–discharge, a high power density, and a long cycle life, have therefore attracted attention as energy-storage components for wearable health-monitoring devices, IoT sensors, and on-chip power supplies [1,2].

MSCs are classified into sandwich-type stacked architectures and in-plane interdigitated architectures. Because in-plane MSCs arrange the positive and negative electrodes alternately in the same plane, no separator is required, and the short ion-transport path between the electrode fingers provides a low ion-transfer resistance and a favorable rate capability. In addition, they are easy to miniaturize and package, which is advantageous for direct integration into thin and flexible electronic devices [3,4].

Carbon-based materials such as graphene oxide (GO), porous graphene, and carbon nanotubes (CNTs) have been widely studied as MSC electrode materials. Reduced graphene, however, is prone to restacking through π–π interactions and van der Waals forces, which decreases the effective surface area accessible to the electrolyte, while residual oxygen functional groups can degrade the electrical conductivity [5,6]. CNTs are similarly limited by bundling, a restricted effective surface area, and high purification costs. Moreover, such carbon-based electrodes generally require binders, current collectors, and multi-step solution processing.

Laser-induced graphene (LIG) is a porous carbon electrode that can overcome these processing limitations. When a CO_2_ laser is irradiated onto a polymer substrate such as polyimide (PI), a localized photothermal reaction cleaves C–O, C=O, and C–N bonds and rearranges the carbon skeleton into an sp^2^-carbon-rich structure. At the same time, the gases evolved during pyrolysis generate a three-dimensional porous network, so that electrodes can be patterned directly without a separate binder or current collector [5,7,8]. In particular, under high energy-input conditions, sheet-like LIG can transform into vertically aligned laser-induced graphene fibers (LIGFs), and LIGF–LIG composite structures can deliver a higher areal capacitance than LIG-only electrodes by extending the external surface area [9,10].

Following the nomenclature established in the original report of this material [7] and in a subsequent review [8], the term “laser-induced graphene” is used here to denote a class of three-dimensional porous graphitic carbon formed by laser-induced pyrolysis of a polymer precursor. It does not imply a defect-free monolayer or few-layer graphene, and the multilayer, partially disordered character of the material is explicitly reflected in the Raman analysis presented in Section 3.2.

The morphology, graphitization quality, and electrical conductivity of LIG electrodes depend strongly on processing parameters such as the laser power, scan speed, pulse density, and focal position [11,12,13]. Previous studies have focused mainly on LIG formation and property changes as functions of laser power and scan speed [11,13]. Defocusing has been established as an effective means of tuning LIG morphology, and previous studies have reported fluence-dependent morphological regimes and fiber formation under high energy input [12,14,15,16,17]. What has been addressed less often is the translation of these morphological regimes into device-level performance: studies that place a defocusing series on a calculated fluence scale and evaluate the resulting electrodes as complete interdigitated MSCs under identical electrolyte, geometry, and area-normalization conditions are limited.

In this work, PI-based LIG/LIGF electrodes were fabricated by varying the defocusing distance of a CO_2_ laser from 0 to 3 mm. Baseline conditions were first selected by evaluating the laser power and scan speed, after which the changes in surface morphology, Raman characteristics, sheet resistance, patterning fidelity, and surface wettability with defocusing were analyzed. Each electrode was then applied to an in-plane interdigitated MSC, and the electrochemical performance and the response under a single bent state were compared, in order to relate the structure, properties, and performance of the LIG electrodes to the defocusing condition. The four conditions were placed on a calculated fluence scale, and their surface chemistry, cross-sectional structure, and porosity were related to the device performance.

## 2. Materials and Methods

### 2.1. Materials

A 75-µm-thick polyimide film (PI film, DuPont, Wilmington, DE, USA) was used as the substrate. Device assembly used insulating polyimide tape (PI tape, 65 µm thick, 3M, Maplewood, MN, USA) and copper tape (Cu tape, Ted Pella Inc., Redding, CA, USA) for current collection. The gel electrolyte was prepared from poly(vinyl alcohol) (PVA, M_w_ 85,000–124,000 g mol^−1^, 87–89% hydrolyzed, Sigma-Aldrich, St. Louis, MO, USA), sulfuric acid (H_2_SO_4_, ≥98%, Sigma-Aldrich, St. Louis, MO, USA), and deionized water. Silver paste (ELCOAT P-100, CANS, Tokyo, Japan) was used to form the current-collecting contacts, and ethanol (≥99.5%, Samchun Pure Chemical Co., Ltd., Pyeongtaek, Republic of Korea) was used for substrate cleaning. All reagents were used as received without further purification.

### 2.2. Fabrication of the LIG Electrodes

**Figure 1:**
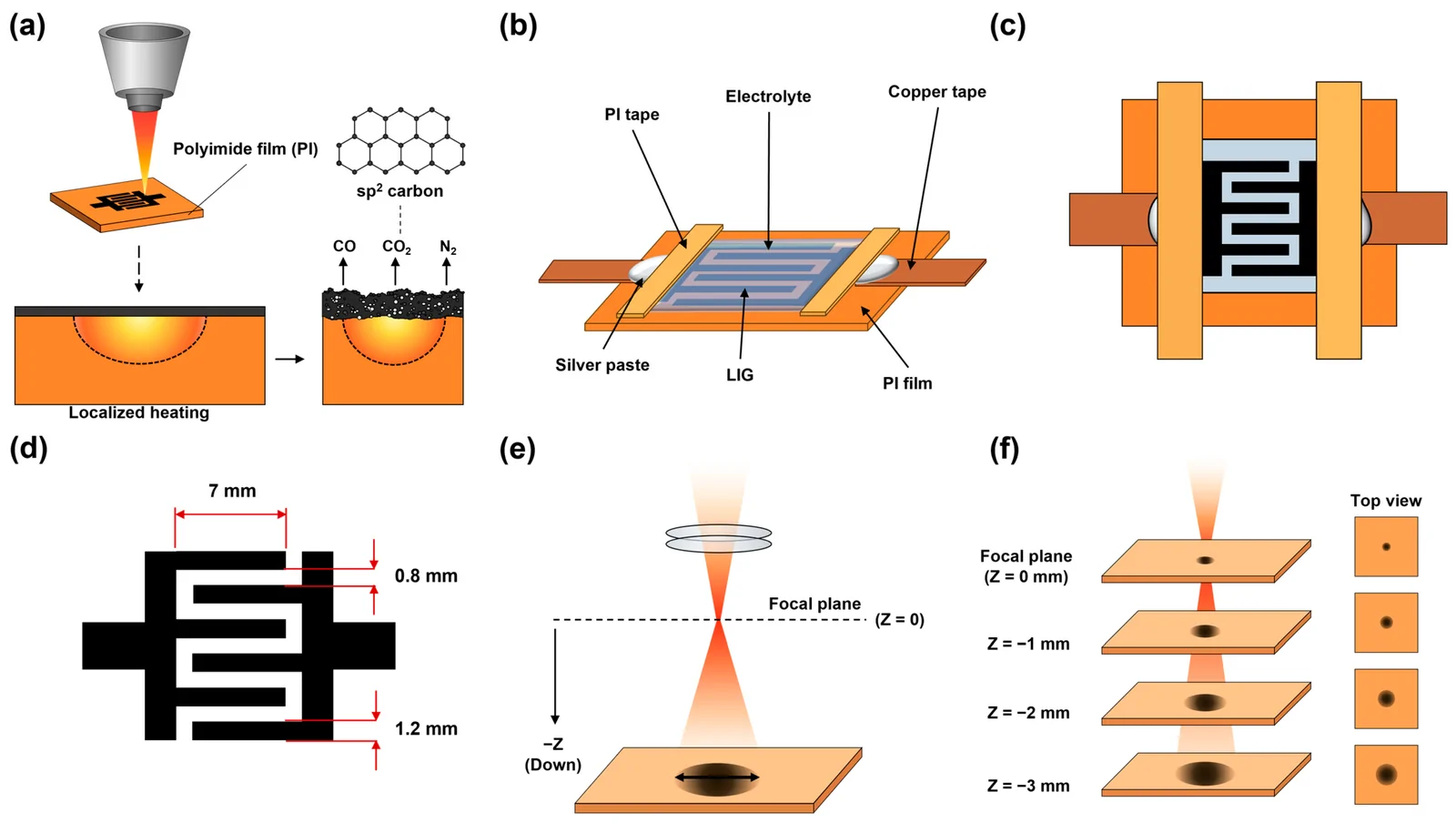
Schematic of the fabrication process for the defocusing-based LIG MSCs. (**a**) Formation of LIG on the PI film surface by CO_2_ laser irradiation. (**b**,**c**) MSC assembly shown in (**b**) three-dimensional and (**c**) top views, including formation of the silver paste and Cu tape current collectors, insulation with PI tape, and application of the gel electrolyte. (**d**) Design of the in-plane interdigitated electrode. (**e**) Focusing geometry of the laser beam and definition of the defocusing distance. (**f**) Enlargement of the laser spot with increasing defocusing distance, with the corresponding top views.

The PI films were ultrasonically cleaned in ethanol and in deionized water for 15 min each and then dried in air for 4 h. LIG was subsequently formed directly on the PI surface in air using a CO_2_ laser marking system (VLS3.60DT, Universal Laser Systems, Scottsdale, AZ, USA; maximum power 50 W, wavelength 9.3 µm; high-power-density focusing optics with a 2.0-inch focal length) (Figure 1a).

The pulse density was fixed at 1000 pulses per inch (PPI); the laser power and scan speed were then evaluated sequentially to select the baseline conditions, and the defocusing distance was adopted as the key processing variable. In this system, PPI denotes the number of pulses per unit travel distance, so that at 1000 PPI the pulse-center spacing remains 25.4 µm regardless of the scan speed. Because the laser spot size is also constant for a given focal condition, the geometric overlap between successive pulses is likewise independent of the scan speed. Changes in scan speed therefore affect the nominal energy input per unit length (*P*/*v*) and the thermal history rather than the pulse-center spacing. At the baseline conditions of 5.0 W and 46 mm s^−1^, the nominal linear energy input is 0.109 J mm^−1^.

The defocusing distance was set by translating the substrate downwards from the focal plane by 0, 1, 2, and 3 mm, and the electrodes produced under these conditions were designated LIGF, LIG-1, LIG-2, and LIG-3, respectively (Figure 1f). Defocusing changes the laser spot and the energy distribution at the substrate surface [8,14,16].

To express the four defocusing conditions on a physically transferable scale, the beam parameters were determined experimentally under the same processing conditions used for the electrodes. Single lines were written on the PI film at 5.0 W, 46 mm s^−1^, and 1000 PPI, and the carbonized line width was measured at defocusing distances of 0, 1, 2, and 3 mm (*n* = 5 per condition) using a digital microscope (Emspira 3, Leica Microsystems, Wetzlar, Germany). Because carbonization proceeds only where the local fluence exceeds a threshold, the diameter of the carbonized region is given by

(1) $$D\left(z\right)=2w(z)\sqrt{\frac{1}{2}ln(\frac{F_{0}(z)}{F_{th}})}$$

where *w*(*z*) = *w*_0_[1 + (*z*/*z*_R_)^2^]^1/2^ is the effective beam radius at a defocusing distance *z*, *F*_0_(*z*) = 2 *E*_p_/[π*w*(*z*)^2^] is the peak fluence per pulse, *E*_p_ is the pulse energy, and *F*th is the carbonization threshold fluence. The pulse energy was obtained as *E*_p_ = *P*/*f*, where *P* is the laser power and *f* = *v*/*p* is the pulse repetition rate determined by the scan speed *v* and the pulse pitch *p* = 25.4 µm at 1000 PPI. With *w*_0_, *z*_R_, and *F*th treated as effective fitting parameters, fitting Equation (1) to the measured line widths yielded *w*_0_ = 102.5 µm, *z*_R_ = 1.13 mm, and *F*th = 1.42 J cm^−2^. Because these parameters describe the extent of the carbonized region rather than the optical beam, they include the contribution of lateral heat diffusion, and the effective spot is considerably larger than the nominal focal spot of the focusing optics, which is on the order of a few tens of micrometers. The resulting spot diameters and peak fluences are listed in Table 1.

### 2.3. Fabrication of the MSCs

The in-plane interdigitated electrodes consisted of eight finger electrodes with a designed finger width, finger gap, and effective length of 1.2, 0.8, and 7 mm, respectively, giving a designed total active area of 0.672 cm^2^ (Figure 1d). For current collection, silver paste was applied to the electrode ends and dried at 60 °C for 10 min to form a conductive film, after which Cu tape was attached and the exposed regions were insulated with PI tape (Figure 1b,c).

The PVA/H_2_SO_4_ gel electrolyte was prepared by dissolving 5 g of PVA in 50 mL of deionized water under stirring at 90 °C for 4 h and then adding 2.5 mL of H_2_SO_4_. Then, 0.3 mL of the electrolyte was applied onto the electrodes and allowed to infiltrate in a vacuum chamber for 2 h to complete the MSC [18].

### 2.4. Characterization

The surface morphology of the electrodes was observed using an ultrahigh-resolution scanning electron microscope (SEM, Gemini 560, Zeiss, Oberkochen, Germany). Raman spectra were acquired at room temperature with a 532 nm laser using a Raman spectrometer (RAMANtouch, Nanophoton Inc., Osaka, Japan). The sheet resistance was measured at room temperature using a four-point probe (CMT-1000MP, AIT Co., Ltd., Suwon, Republic of Korea); five measurements were taken per specimen, and the mean and standard deviation were calculated. Changes in the heat-affected zone with defocusing were compared by measuring the finger width with a digital microscope (Emspira 3, Leica Microsystems, Wetzlar, Germany). The surface wettability was evaluated from the static contact angle of a 5 µL deionized-water droplet using a contact-angle analyzer (Smartdrop Plus, Femtobiomed Co., Ltd., Seongnam, Republic of Korea). Five measurements were taken at different positions on each specimen, and the mean and standard deviation were calculated.

The cross-sectional morphology was examined with the same SEM at an accelerating voltage of 3 kV using a secondary-electron detector (Zeiss, Oberkochen, Germany), and the thickness of the carbonized layer was measured at ten positions on each image. The elemental composition was analyzed by energy-dispersive X-ray spectroscopy (EDS, Oxford Instruments, Abingdon, UK) attached to the SEM at an accelerating voltage of 5 kV and by X-ray photoelectron spectroscopy (XPS, Quantera II, ULVAC-PHI, Chigasaki, Japan) using monochromatic Al Kα radiation (1486.6 eV) with a beam diameter of 100 µm, a take-off angle of 45°, and dual-beam charge neutralization. Survey and high-resolution spectra were acquired at pass energies of 280 and 55 eV, respectively, on the as-prepared surfaces without sputter cleaning. Atomic concentrations were determined from the high-resolution C 1s, N 1s, and O 1s spectra using the instrument sensitivity factors; binding energies were referenced to the sp^2^ C 1s component at 284.5 eV, and the C 1s spectra were fitted after Shirley background subtraction with six Gaussian–Lorentzian components assigned to sp^2^ C=C (284.5 eV), C–C (285.1 eV), C–O (286.3 eV), C=O (287.4 eV), O–C=O (288.8 eV), and the π–π* shake-up satellite (approximately 290.7 eV), using a common width for the C–C, C–O, C=O, and O–C=O components. Surface functional groups were also examined by attenuated total reflectance Fourier-transform infrared spectroscopy (ATR-FTIR, Cary 670 spectrometer with a Cary 620 microscope, Agilent Technologies, Santa Clara, CA, USA) with a pristine PI film as a reference. The specific surface area and pore structure of LIG-2 and LIG-3 were evaluated from N_2_ adsorption–desorption isotherms measured at 77 K (ASAP 2425, Micromeritics Instrument Corp., Norcross, GA, USA) using carbon scraped from the PI substrate after degassing at 200 °C. The Brunauer–Emmett–Teller (BET) surface area was calculated in the relative pressure range of 0.01–0.08, selected according to the Rouquerol consistency criteria; the micropore area and volume were obtained by the t-plot method with the Harkins–Jura thickness equation, and the pore size distribution was derived from the adsorption branch using the Barrett–Joyner–Halenda (BJH) method. The total cross-sectional thickness was additionally determined from the outer boundaries of the sample in the same images.

### 2.5. Electrochemical Measurements

Cyclic voltammetry (CV), galvanostatic charge–discharge (GCD), and electrochemical impedance spectroscopy (EIS) were performed in a two-electrode configuration using an electrochemical analyzer (WISEIS-1200 premium, WizMAC Co., Ltd., Daejeon, Republic of Korea). The voltage window was set to 0–0.8 V, and EIS was carried out from 100 kHz to 0.1 Hz at the open-circuit potential with an AC amplitude of 10 mV. Cycling stability was evaluated by repeating CV 2000 times at a scan rate of 50 mV s^−1^ over 0–0.8 V and tracking the retention of the capacitance calculated from the integrated CV area. Flexibility was assessed by measuring CV while the device was held at a bending angle of 180° and comparing the result with that obtained in the flat state.

The areal capacitance (*C*_A_), areal energy density (*E*_A_), and areal power density (*P*_A_) were calculated using Equations (2)–(4), respectively [19].

(2) $$C_{A}=\frac{i\times ∆t}{S∆V}$$

(3) $$E_{A}=\frac{C_{A}\times {∆V}^{2}}{2\times 3600}$$

(4) $$P_{A}=\frac{E_{A}\times 3600}{∆t}$$

where *i* is the discharge current (mA), Δ*t* is the discharge time (s), Δ*V* is the applied voltage window (V), taken as 0.8 V in this study without IR-drop correction, and *S* is the total active area of the positive and negative electrodes (cm^2^) [4]. *C*_A_, *E*_A_, and *P*_A_ of each device were calculated using the carbonized area estimated from the measured finger width of the corresponding electrode as *S*. At 0.05 mA cm^−2^, the applied currents were 0.04312 mA for LIGF, 0.04480 mA for LIG-1, 0.04648 mA for LIG-2, and 0.04592 mA for LIG-3. Higher current densities of 0.10–0.25 mA cm^−2^ were obtained by applying integer multiples of these base currents, and the current density and areal capacitance were calculated using the same estimated-area basis. The units of the areal capacitance, areal energy density, and areal power density obtained from Equations (2)–(4) are mF cm^−2^, mWh cm^−2^, and mW cm^−2^, respectively; energy densities are reported in µWh cm^−2^ in the text.

## 3. Results and Discussion

### 3.1. Selection of the Baseline Laser Power and Scan Speed

**Figure 2:**
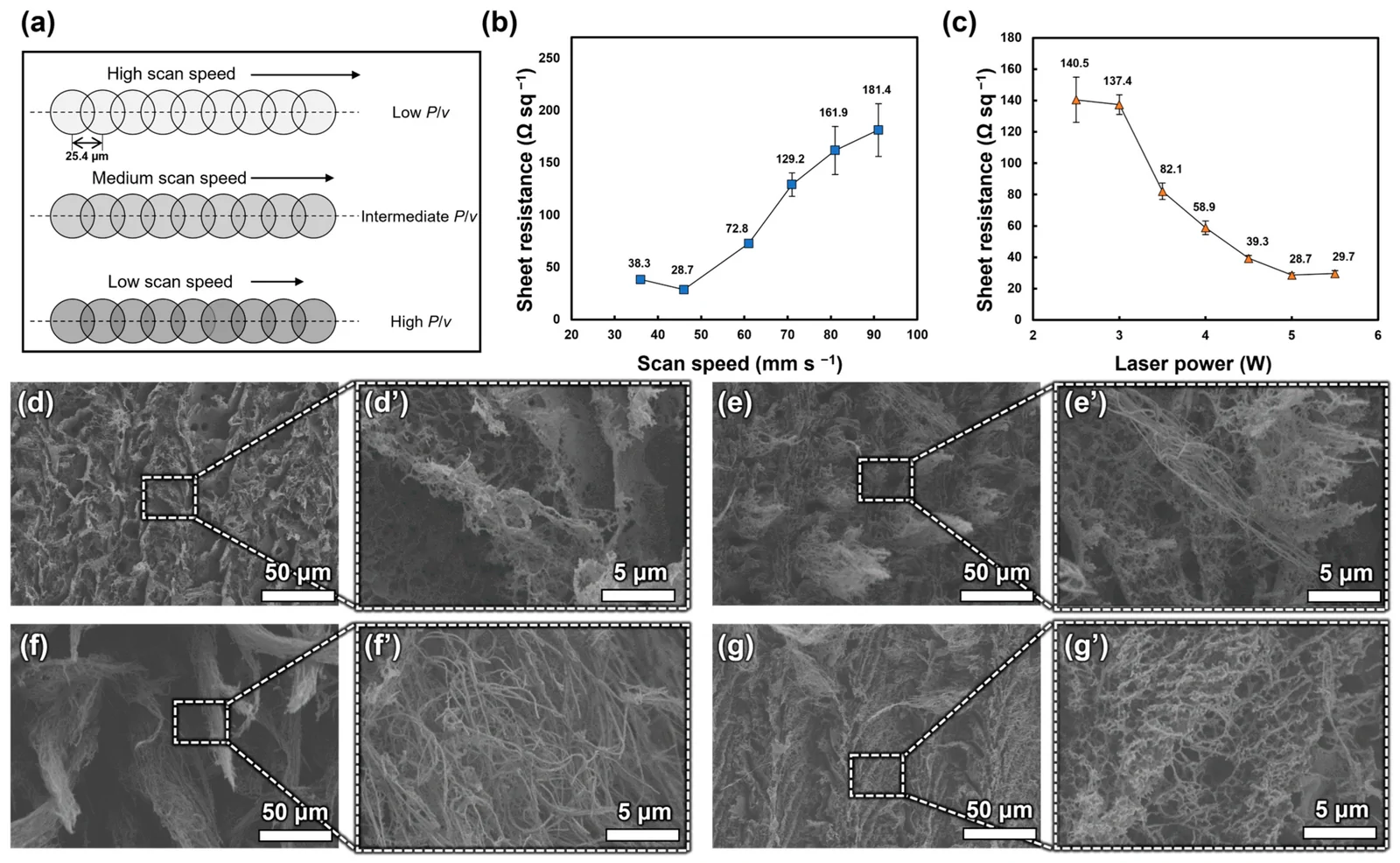
Electrical properties and surface microstructure of LIG as functions of the laser processing parameters. (**a**) Schematic of the pulse-center spacing (25.4 µm), which remains constant at a pulse density of 1000 PPI, and of the variation in the nominal linear energy input (*P*/*v*) with scan speed. (**b**) Sheet resistance as a function of scan speed at a laser power of 5.0 W. (**c**) Sheet resistance as a function of laser power at a scan speed of 46 mm s^−1^. (**d**,**d′**) 4.0 W, (**e**,**e′**) 4.5 W, (**f**,**f′**) 5.0 W, and (**g**,**g′**) 5.5 W: SEM images of the LIG surfaces formed at a scan speed of 46 mm s^−1^ under the focal-plane condition. Panels (**d**–**g**) are low-magnification images, and panels (**d′**–**g′**) are high-magnification images of the regions outlined by the dashed boxes.

The fabrication process and electrode design of the defocusing-based LIG/LIGF MSCs are summarized in Figure 1. LIG or LIGF was formed directly on the PI surface by CO_2_ laser irradiation (Figure 1a); current-collecting contacts were then formed with silver paste and Cu tape, the exposed regions were insulated with PI tape, and the PVA/H_2_SO_4_ gel electrolyte was applied to complete the MSC (Figure 1b,c). The electrodes were designed as an in-plane interdigitated structure consisting of eight finger electrodes with a finger width, finger gap, and effective length of 1.2, 0.8, and 7 mm, respectively (Figure 1d). Because the morphology and sheet resistance of LIG are affected by the laser power, scan speed, pulse density, and focal position [11,12,13], the pulse density was fixed at 1000 PPI, baseline conditions for the scan speed and laser power were selected (Figure 2a–c), and the electrode characteristics were then analyzed as functions of the defocusing distance (Figure 1e,f).

Figure 2a shows that, with the pulse density fixed at 1000 PPI, the pulse-center spacing remains constant at 25.4 µm. Because the laser power (5.0 W) and the focal-plane condition were fixed in this experiment, changes in scan speed were expressed as changes in the nominal linear energy input (*P*/*v*) rather than as changes in the pulse-center spacing or in the geometric overlap of the laser spots; *P*/*v* therefore increases as the scan speed decreases.

Figure 2b shows the variation in sheet resistance with scan speed at a laser power of 5.0 W. As the scan speed decreased from 91 to 46 mm s^−1^, the sheet resistance decreased monotonically from 181.4 Ω sq^−1^ (91 mm s^−1^) to 161.9 Ω sq^−1^ (81 mm s^−1^), 129.2 Ω sq^−1^ (71 mm s^−1^), 72.8 Ω sq^−1^ (61 mm s^−1^), and 28.7 Ω sq^−1^ (46 mm s^−1^). At 36 mm s^−1^, however, it increased again to 38.3 Ω sq^−1^. This increase may be related to changes in the LIG structure caused by excessive energy input, and is consistent with previous reports that excessive or repeated laser irradiation can affect the defect structure and morphology of the carbon skeleton [9,10,12]. The scan speed of 46 mm s^−1^, which gave the lowest sheet resistance within the measured range, was therefore selected as the baseline for the subsequent experiments. At 102 mm s^−1^, no continuous conductive film was formed and the sheet resistance could not be measured.

**Figure 3:**
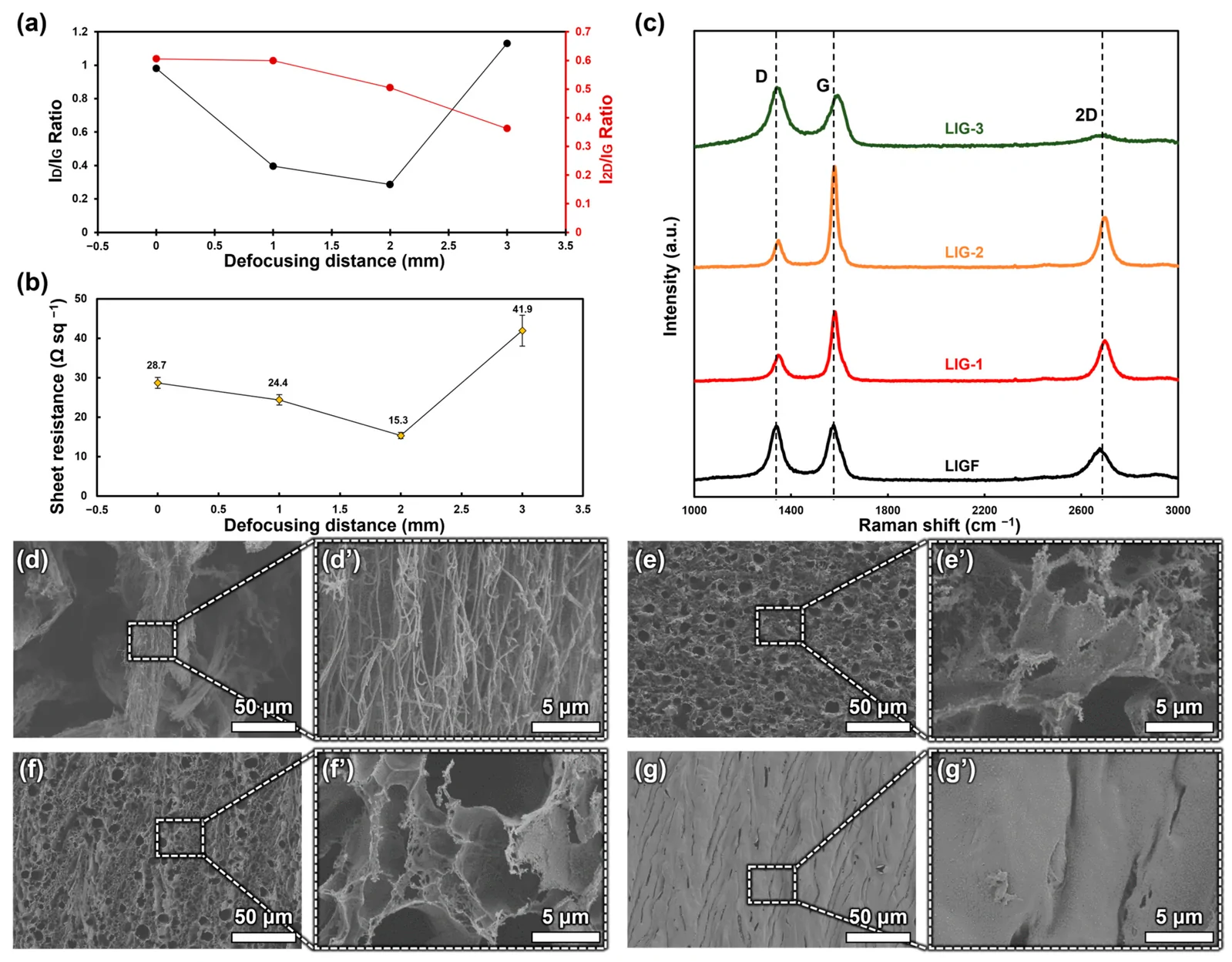
Structural and electrical characteristics and surface morphology of the LIG/LIGF electrodes as functions of the defocusing distance (laser power 5.0 W, scan speed 46 mm s^−1^). (**a**) I_D_/I_G_ and I_2D_/I_G_ ratios versus defocusing distance (0–3 mm). (**b**) Sheet resistance versus defocusing distance. (**c**) Raman spectra (532 nm) of LIGF, LIG-1, LIG-2, and LIG-3. (**d**,**d′**) LIGF (0 mm), (**e**,**e′**) LIG-1 (1 mm), (**f**,**f′**) LIG-2 (2 mm), and (**g**,**g′**) LIG-3 (3 mm): SEM images of the electrode surfaces. Panels (**d**–**g**) are low-magnification images, and panels (**d′**–**g′**) are high-magnification images of the regions outlined by the dashed boxes.

Figure 2c shows the results obtained when the laser power was varied from 2.5 to 5.5 W at a fixed scan speed of 46 mm s^−1^. The sheet resistance was high at 2.5 and 3.0 W (140.5 and 137.4 Ω sq^−1^, respectively), which is attributed to these conditions lying near the carbonization threshold of PI [7,20], so that a sufficiently conductive graphene network was not formed. As the laser power increased above 3.5 W, the sheet resistance decreased to 82.1 (3.5 W), 58.9 (4.0 W), and 39.3 Ω sq^−1^ (4.5 W), reaching a minimum of 28.7 Ω sq^−1^ at 5.0 W. At 5.5 W, a value of 29.7 Ω sq^−1^ was measured, comparable to that at 5.0 W within the standard deviation. A laser power of 5.0 W, which yielded the minimum sheet resistance and was the lower of the two powers with comparable sheet-resistance values, was therefore selected as the baseline. The Raman spectra, sheet resistance, and Raman intensity ratios as functions of the defocusing distance (Figure 3a–c) are discussed in Section 3.2.

To compare the surface microstructures obtained at different laser powers, the LIG formed at 4.0–5.5 W was examined by SEM (Figure 2d–g′). At 4.0 W, a porous sheet-like structure was predominant and fibrous features were confined to limited regions (Figure 2d,d′). At 4.5 W, fibrous structures and LIGF bundles became more abundant (Figure 2e,e′), and at 5.0 W, densely developed fibers several micrometers or more in length dominated the observed area (Figure 2f,f′). At 5.5 W, in contrast, the fine continuous fibers decreased, and short, thick strands coexisted with coarse pores (Figure 2g,g′).

The transition from a sheet-like to a fibrous structure with increasing laser power is consistent with the morphological changes in LIG reported previously [9,10]. Because fibrous structures were predominant at 5.0 W, 46 mm s^−1^, and the focal condition, the electrode fabricated at the focal plane (0 mm) was designated LIGF. Figure 2f and Figure 3d correspond to different observation areas of the same processing condition, and the distribution and alignment of the fibers differed between local positions. The laser power and scan speed were subsequently fixed at 5.0 W and 46 mm s^−1^, and the changes in morphology and properties with defocusing were compared.

### 3.2. Carbonization Quality and Electrical Properties Versus Defocusing Distance

Using the selected laser power of 5.0 W and scan speed of 46 mm s^−1^, the defocusing distance was varied from 0 to 3 mm, and the Raman intensity ratios, sheet resistance, and Raman spectra of the resulting LIGF, LIG-1, LIG-2, and LIG-3 electrodes were compared (Figure 3a–c). All electrodes exhibited a D band at approximately 1350 cm^−1^, a G band at approximately 1580 cm^−1^, and a 2D band at approximately 2700 cm^−1^. The D band is a disorder-related mode activated by defects and edges, the G band corresponds to the E_2g_ vibrational mode of sp^2^ carbon, and the 2D band is associated with double-resonance scattering [7,8,21].

In Figure 3a, I_D_/I_G_ decreased from 0.98 for LIGF to 0.40 for LIG-1 and 0.29 for LIG-2, and then increased to 1.13 for LIG-3, showing a V-shaped trend similar to that of the sheet resistance. I_2D_/I_G_ decreased monotonically with increasing defocusing distance, from 0.61 to 0.60, 0.50, and 0.36. The lowest I_D_/I_G_ of LIG-2 indicates that the D-band-related defect signal was relatively low among the four conditions. It should be noted, however, that the Raman intensity ratios of laser-induced graphene can vary with the laser wavelength and the processing conditions [11,13], and that the relationship between I_D_/I_G_ and the degree of disorder is not necessarily monotonic for highly disordered carbon [21]. Moreover, because the number of graphene layers in porous LIG cannot be quantified from I_2D_/I_G_ alone [22], the two intensity ratios were used in this study only as relative indicators for comparing the Raman characteristics among the conditions.

In Figure 3b, the sheet resistance decreased from 28.7 Ω sq^−1^ for LIGF to 24.4 Ω sq^−1^ for LIG-1 and 15.3 Ω sq^−1^ for LIG-2, and then increased again to 41.9 Ω sq^−1^ for LIG-3, following a V-shaped trend. LIG-3 in particular showed a larger standard deviation (±3.9 Ω sq^−1^) than the other conditions. Within the present experimental range, the 2 mm defocusing condition was therefore the most favorable for producing a low and uniform sheet resistance.

This trend was also reflected in the Raman spectra of Figure 3c. LIG-2 exhibited the weakest D band and a distinct G band among the four conditions, whereas LIG-3 showed a relatively strong D band and a weak 2D band. The relative intensity of the 2D band also decreased monotonically with increasing defocusing distance, consistent with the variation in I_2D_/I_G_ shown in Figure 3a.

**Figure 4:**
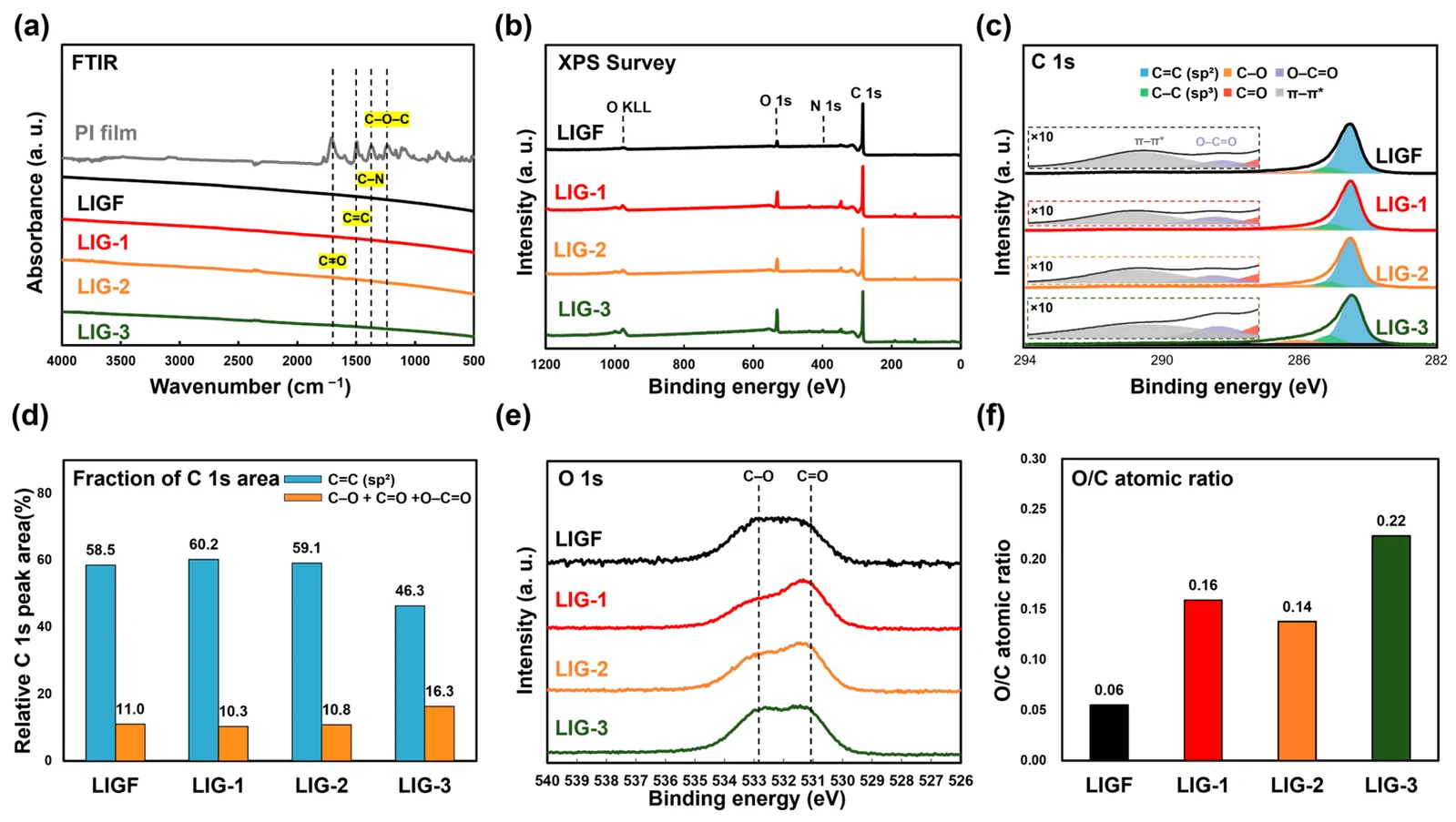
Chemical characterization of the LIG/LIGF electrodes. (**a**) ATR-FTIR spectra of the pristine PI film (intensity magnified three times) and the electrodes, vertically offset for clarity; the dashed lines mark the characteristic PI bands at 1711 (imide C=O), 1496 (aromatic C=C), 1371 (imide C–N), and 1234 cm^−1^ (C–O–C). (**b**) XPS survey spectra. (**c**) High-resolution C 1s spectra (solid lines) and fitted components (shaded areas); the insets show ×10 magnified views of the 287.8–293.8 eV region with the background-subtracted data (open circles) and the fitted envelope (black line). (**d**) Relative peak areas of the sp^2^ C=C component and of the oxygen-bonded components (C–O + C=O + O–C=O), expressed as percentages of the total fitted C 1s area. (**e**) High-resolution O 1s spectra; the dashed lines mark the typical binding energies of C=O (531.5 eV) and C–O (533 eV). (**f**) Surface O/C atomic ratio calculated from the high-resolution spectra. The XPS spectra in (**b**,**c**,**e**) are normalized and vertically offset.

Taken together, the Raman intensity ratios and the sheet resistance varied jointly with the defocusing distance, and both indicators were lowest for LIG-2. This shows that, within the present test range, LIG-2 simultaneously exhibited the lowest sheet resistance and the lowest I_D_/I_G_. The elemental composition and surface chemistry were further examined to confirm the carbonization state (Figure 4). In the ATR-FTIR spectra (Figure 4a), the characteristic bands of the pristine PI at 1711 (imide C=O), 1496 (aromatic C=C), 1371 (imide C–N), and 1234 cm^−1^ (C–O–C) were absent for all four electrodes, and no distinct absorption attributable to oxygen-containing functional groups was resolved, indicating essentially complete conversion of the PI into an sp^2^-bonded carbon network. EDS, which probes a depth of several hundred nanometers at 5 kV, gave carbon contents of 98.1–98.6 at%, oxygen contents of 1.0–1.5 at%, and nitrogen contents below 0.5 at% for all conditions. XPS, which probes the outermost few nanometers, gave surface compositions (normalized to C, N, and O) of 94.5, 85.5, 87.0, and 80.0 at% C, 5.2, 13.6, 12.0, and 17.9 at% O, and 0.3, 0.9, 1.0, and 2.1 at% N for LIGF, LIG-1, LIG-2, and LIG-3, respectively, so that the surface oxygen and nitrogen contents were lowest for LIGF and highest for LIG-3 (Figure 4b,f). Deconvolution of the C 1s spectra (Figure 4c,d) showed that sp^2^ carbon was the dominant component in LIGF, LIG-1, and LIG-2 (58.5–60.2% of the total C 1s area), with oxygen-bonded carbon (C–O, C=O, and O–C=O) accounting for 10.3–11.0%, whereas LIG-3 exhibited a lower sp^2^ fraction (46.3%) and a higher fraction of oxygen-bonded carbon (16.3%). Although the O 1s spectra (Figure 4e) were not deconvoluted, the envelopes of LIG-1 and LIG-2 peaked near 531.5 eV, typical of C=O, whereas that of LIG-3 showed comparable intensities near 531.5 and 533 eV, the latter typical of C–O, which is qualitatively consistent with its higher fraction of oxygen-bonded carbon. Thus, the carbonized layers consisted essentially of carbon below the outermost surface, whereas the surface of LIG-3 retained more oxygen- and nitrogen-containing species, indicating that the conversion at its surface was less complete. The survey spectra also showed weak Ca and P signals, which are attributed to inorganic additives in the commercial PI film; the atomic compositions reported here were therefore normalized to C, N, and O.

### 3.3. Morphological Evolution with Defocusing

The changes in the surface microstructure of the electrodes with defocusing distance are shown in Figure 3d–g′. Low- and high-magnification SEM images of each condition were compared in order to analyze the pore distribution and the fine morphology.

In LIGF, fabricated at the focal plane, raised bands several tens of micrometers wide formed along the laser scan direction, while the surrounding regions appeared relatively dark and smooth (Figure 3d). The high-magnification image reveals long, thin fibers several hundred nanometers in diameter and several micrometers in length, densely developed on the raised regions (Figure 3d′).

In LIG-1, obtained at a defocusing distance of 1 mm, fibrous structures decreased and circular open pores approximately 5–15 µm in diameter were distributed across the surface (Figure 3e). The high-magnification image shows wrinkled sheet-like structures along the pore walls, with relatively smooth regions remaining between the pores (Figure 3e′).

In LIG-2, obtained at 2 mm, pores ranging from a few micrometers to 20 µm were densely distributed over the entire surface (Figure 3f). At high magnification, the open pores were partitioned by wall structures and formed an open-cell-like network that was interconnected within the observed plane (Figure 3f′). This structure was clearly distinguishable from that of LIG-1, in which pores were distributed between smooth regions; LIG-2 also simultaneously exhibited the lowest sheet resistance (15.3 Ω sq^−1^) and the lowest I_D_/I_G_ (0.29).

In LIG-3, obtained at 3 mm, circular pores were rarely observed, and a smooth surface with wave-like undulations along the scan direction extended over a wide area (Figure 3g). Even at high magnification, a smooth surface with narrow cracks was observed rather than a porous framework (Figure 3g′). LIG-3 exhibited the highest sheet resistance (41.9 Ω sq^−1^) and the highest I_D_/I_G_ (1.13), together with the largest measurement scatter in sheet resistance.

Thus, as the defocusing distance increased, the surface morphology changed from a fibrous structure (LIGF) to a porous structure (LIG-1), an open-cell-like pore network (LIG-2), and finally a smooth structure with few pores (LIG-3). In particular, the characteristic pore network of LIG-2 was accompanied by the lowest sheet resistance and the lowest I_D_/I_G_. These morphological changes are interpreted in terms of the variation in laser spot size and peak fluence per pulse with defocusing (Figure 1f and Table 1). At the focal plane, the spot size is smallest and the peak fluence per pulse is highest (Table 1), which is considered to promote rapid localized pyrolysis and the formation of fibrous carbon. In such fibrous structures, however, contact between the fibers is limited, so that continuous in-plane conduction paths may not be sufficiently established. When the substrate is lowered, the laser spot enlarges and the peak fluence per pulse decreases, whereas the geometric overlap between adjacent irradiated areas increases because the pulse-center spacing remains fixed at 25.4 µm. As a result, the same area can be exposed to multiple laser pulses during a single scan [23], and this thermal history may have contributed to the formation of the continuous pore framework observed at 2 mm. At 3 mm, in contrast, the peak fluence per pulse decreased to only about 1.5 times the carbonization threshold [12,20], and a smooth, pore-scarce surface was formed, which is consistent with the high sheet resistance.

The fluence scale established in Section 2.2 provides a physical basis for these observations. The peak fluence per pulse decreases by approximately one order of magnitude across the series, from 16.7 J cm^−2^ at 0 mm to 2.1 J cm^−2^ at 3 mm, while the number of pulses overlapping along the scan direction increases from 8 to 23 (Table 1). The carbonized line width does not increase monotonically with the defocusing distance but reaches a maximum at 2 mm, which is a direct consequence of threshold-limited carbonization: the beam expands with increasing defocusing distance while the peak fluence falls, and the width of the supra-threshold region is maximized where these two effects balance. The carbonized finger widths of the interdigitated electrodes show a similar trend, increasing up to 2 mm and remaining nearly unchanged at 3 mm (Section 3.4). This mapping is consistent with the fluence-dependent morphological regimes reported for laser-induced carbon on polyimide. Abdulhafez et al. [12] identified successive transitions from an isotropic porous morphology to anisotropic cellular networks and then to aligned woolly nanofibers with increasing fluence, and found the anisotropic cellular network to exhibit the highest electrical conductivity and the highest degree of graphitization. In the present series, LIGF, formed at the highest fluence, shows the fibrous morphology, whereas LIG-2 shows an open-cell-like network together with the lowest sheet resistance and the lowest I_D_/I_G_ ratio. The optimum identified at 2 mm therefore corresponds to a morphological regime rather than to a distance specific to the present setup. The carbonization threshold obtained here (1.42 J cm^−2^ per pulse) is lower than the single-pulse threshold of approximately 5.5 J cm^−2^ reported previously [9,23], which is consistent with the accumulation of 8–23 pulses along the scan direction in the present process. A direct numerical comparison of absolute fluence values with these reports is not attempted, because the wavelength (9.3 versus 10.6 µm), scan speed (46 mm s^−1^ versus 500 mm s^−1^ in ref. [12]), and use of overlapping raster lines differ.

**Figure 5:**
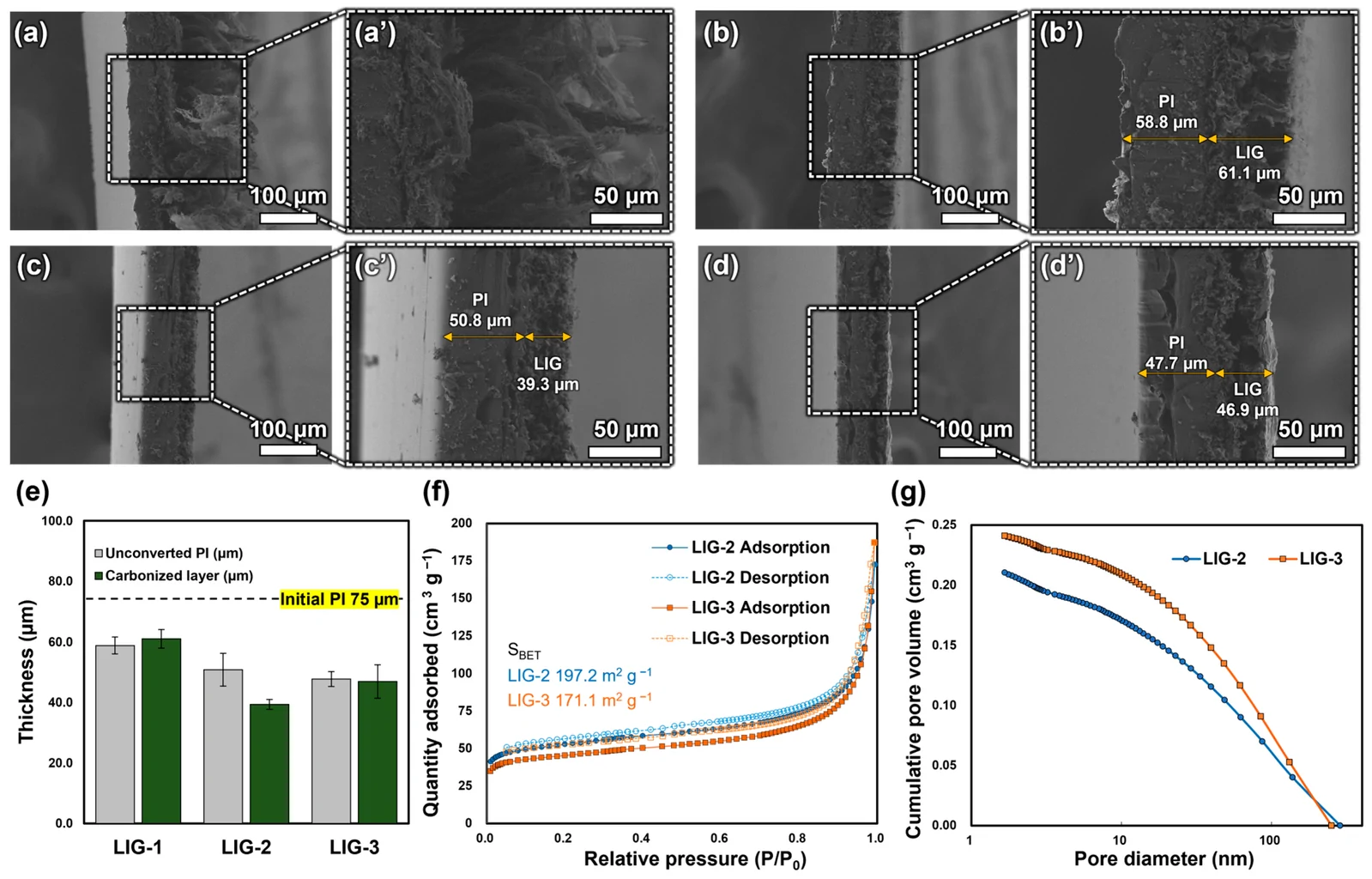
Cross-sectional structure and porosity of the electrodes. (**a**,**a′**) LIGF (0 mm), (**b**,**b′**) LIG-1 (1 mm), (**c**,**c′**) LIG-2 (2 mm), and (**d**,**d′**) LIG-3 (3 mm): cross-sectional SEM images. Panels (**a**–**d**) are low-magnification images, and panels (**a′**–**d′**) are high-magnification images of the regions outlined by the dashed boxes; the arrows in (**b′**–**d′**) indicate the thicknesses of the carbonized layer and the unconverted PI. (**e**) Thickness of the carbonized layer and the unconverted PI for LIG-1, LIG-2, and LIG-3 (mean ± standard deviation, *n* = 10); the dashed line marks the initial PI thickness. (**f**) N_2_ adsorption–desorption isotherms measured at 77 K, with the BET surface areas indicated, and (**g**) BJH cumulative pore volume derived from the adsorption branch for LIG-2 and LIG-3.

The cross-sectional SEM images (Figure 5a–d′) show that the porous carbon layer extends continuously from the electrode surface to the underlying PI under all conditions. For LIGF, the carbonized region consisted of a thin porous base covered by fiber bundles that extended more than 100 µm from it, so that a single layer thickness could not be defined; the thickness was therefore quantified only for LIG-1 to LIG-3. Measured at ten positions per condition on the high-magnification images (Figure 5a′–d′,e), the carbonized layer was 61.1 ± 3.1, 39.3 ± 1.6, and 46.9 ± 5.5 µm thick and the unconverted PI was 58.8 ± 2.8, 50.8 ± 5.4, and 47.7 ± 2.4 µm thick for LIG-1, LIG-2, and LIG-3, respectively, so that the carbonized layer was thicker at 3 mm than at 2 mm. Because the laser power, scan speed, and raster line spacing were identical, the energy delivered per unit area was the same for all conditions, and defocusing redistributed this energy over a larger number of lower-fluence pulses rather than reducing it. Relative to the initial PI thickness of 75 µm, the converted depth was 16, 24, and 27 µm for LIG-1, LIG-2, and LIG-3 (Figure 5e), so that the carbonized layer of LIG-1 had expanded to about 3.8 times its converted depth, compared with about 1.6 and 1.7 times for LIG-2 and LIG-3. The larger layer thickness of LIG-1 therefore reflects a greater volumetric expansion, consistent with the large cavities in its cross-section, rather than a deeper conversion. In LIG-1 and LIG-2, the pore network was open to the surface, and in LIG-2 it consisted of fine pores distributed uniformly throughout the layer. In LIG-3, by contrast, a smooth, pore-scarce surface layer covered a porous interior containing large voids, consistent with the pore-scarce surface observed in Figure 3g. The total cross-sectional thickness, measured from the outer surface to the back of the film, was 123, 91, and 95 µm for LIG-1, LIG-2, and LIG-3, that is, 48, 16, and 20 µm larger than the initial film thickness of 75 µm; this comparison does not depend on the position of the PI/LIG boundary and therefore supports the larger volumetric expansion at 1 mm independently of the layer measurement.

Nitrogen physisorption was performed on LIG-2 and LIG-3, which represent the optimal condition and the condition showing the performance decrease, respectively. The N_2_ adsorption–desorption isotherms of LIG-2 and LIG-3 (Figure 5f,g) showed a steep uptake at low relative pressures followed by a hysteresis loop, indicating the coexistence of micropores and mesopores. The BET surface areas were 197.2 and 171.1 m^2^ g^−1^ for LIG-2 and LIG-3, respectively, and micropores accounted for approximately 75% of the surface area in both samples. The total pore volume of LIG-3 (0.290 cm^3^ g^−1^) was slightly larger than that of LIG-2 (0.267 cm^3^ g^−1^); however, LIG-2 contained more micropore volume (0.061 versus 0.052 cm^3^ g^−1^) and more pore volume below 10 nm (0.040 versus 0.032 cm^3^ g^−1^), whereas the additional pore volume of LIG-3 was concentrated in pores larger than 50 nm, which contribute little to the surface area.

Taken together, the carbonized layer of LIG-3 was neither thinner than that of LIG-2 nor densified throughout its thickness, and its internal surface area was of the same order. Together with the compositional results in Section 3.2, these observations show that the lower performance of LIG-3 does not arise from insufficient carbonization of the layer as a whole. Instead, the highest surface oxygen and nitrogen contents and the lowest sp^2^ fraction among the electrodes are consistent with less complete conversion at the surface of LIG-3 under the near-threshold peak fluence at 3 mm. The smooth, pore-scarce surface layer may limit the access of the gel electrolyte to the porous interior. Together with the lower graphitic order indicated by the high I_D_/I_G_ ratio (1.13), this surface structure may contribute to the higher sheet resistance and lower capacitance of LIG-3. This interpretation rests on the combined cross-sectional, compositional, and porosity evidence rather than on a depth-resolved measurement of the surface layer.

### 3.4. Patterning Fidelity and Surface Wettability

**Figure 6:**
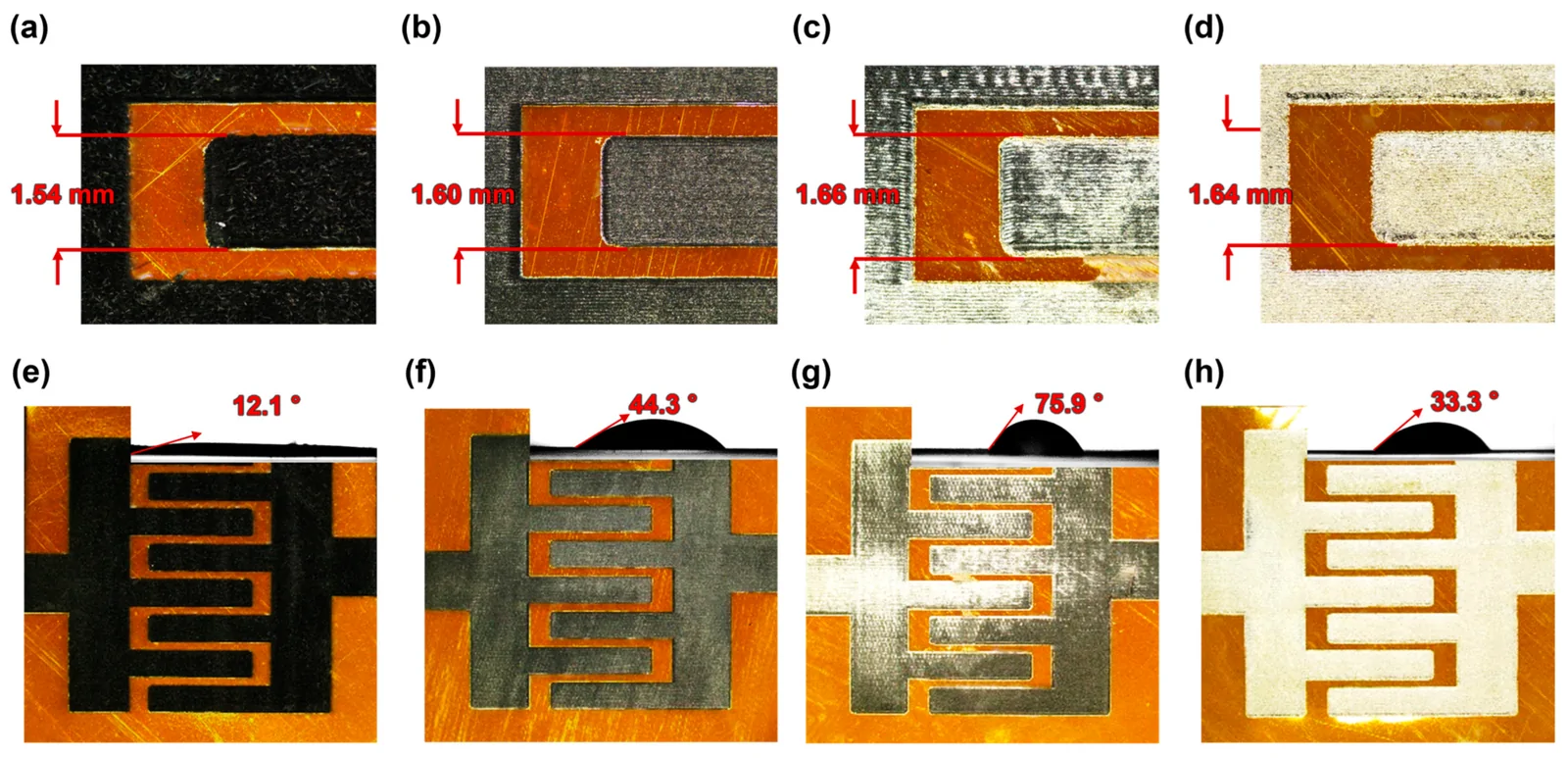
Patterning fidelity and surface wettability of the electrodes for each defocusing condition. (**a**–**d**) Electrode finger widths measured with a digital microscope: (**a**) LIGF, (**b**) LIG-1, (**c**) LIG-2, and (**d**) LIG-3. (**e**–**h**) Optical images of the devices and the corresponding water contact angles (5 µL deionized water): (**e**) LIGF, (**f**) LIG-1, (**g**) LIG-2, and (**h**) LIG-3. The designed finger width is 1.2 mm.

The patterning fidelity as a function of defocusing was evaluated from the electrode finger widths measured with a digital microscope (Figure 6a–d). Against a designed width of 1.2 mm, the measured widths were 1.54 mm for LIGF, 1.60 mm for LIG-1, 1.66 mm for LIG-2, and 1.64 mm for LIG-3. The finger width increased between 0 and 2 mm of defocusing and was similar at 2 and 3 mm. Although the width measurement for LIG-3 may carry a relatively large uncertainty because of its low optical contrast with the unprocessed region, line-width broadening caused by laser irradiation was clearly observed under all conditions.

The measured finger widths of all electrodes were 28.3–38.3% larger than the designed value, and the effective gap between adjacent fingers decreased from the designed value of 0.8 mm to approximately 0.34–0.46 mm. This shows that defocusing affects not only the electrode properties but also the patterning fidelity and resolution of the micropattern [20]. Pre-compensation of the finger width and finger gap is therefore required when a defocusing process is applied to MSC patterning. The lateral broadening per side, obtained as half the difference between the measured and designed finger widths, was 0.170, 0.200, 0.230, and 0.220 mm for LIGF, LIG-1, LIG-2, and LIG-3, respectively. Because the scan path fixes the pattern pitch at 2.0 mm, defocusing redistributes the width and the gap without altering the period, and the design width required to obtain a 1.2 mm carbonized finger can be estimated as 0.86, 0.80, 0.74, and 0.76 mm for the four conditions. The carbonized areas calculated from the measured widths were 0.862, 0.896, 0.930, and 0.918 cm^2^ for LIGF, LIG-1, LIG-2, and LIG-3, respectively, and these values were used to normalize the electrochemical performance in Section 3.5. Here, the carbonized area includes only the finger regions; the carbonized area of the bus bars connected to the current collectors was excluded. For reference, the device footprint area including the inter-finger gaps and the outer margins is approximately 1.12 cm^2^.

The surface wettability was evaluated from the static contact angle of a 5 µL deionized-water droplet (Figure 6e–h). The contact angles were 12.1 ± 2.6° for LIGF, 44.3 ± 7.6° for LIG-1, 75.9 ± 5.7° for LIG-2, and 33.3 ± 14.7° for LIG-3, so that all electrodes presented hydrophilic surfaces with contact angles below 90°. The scatter was considerably larger for LIG-3 (standard deviation 14.7°, with individual values ranging from 19.8° to 55.4°) than for the other electrodes, which is attributed to the spatially non-uniform surface of LIG-3, where a smooth surface skin coexists with narrow cracks (Figure 3g′ and Section 3.3). LIGF in particular showed the lowest contact angle and hence the best wettability among the four electrodes. Such differences can arise from the combined influence of surface chemistry and microstructure [15,24], although the two factors were not quantified separately in this study. Among the porous electrodes, the contact angle decreased as the surface oxygen content increased, from LIG-2 (12.0 at% O) to LIG-1 (13.6 at%) and LIG-3 (17.9 at%) (Section 3.2), consistent with the contribution of oxygen-containing groups to wettability. LIGF, however, showed the smallest contact angle despite the lowest surface oxygen content (5.2 at%), indicating that its wettability was governed primarily by the fibrous morphology rather than by the surface chemistry.

The contact angle did not vary monotonically with defocusing distance, and the largest value was measured for LIG-2, which had the lowest sheet resistance. Within the present experimental range, therefore, no simple correlation was found between surface wettability and sheet resistance. Moreover, because the static water contact angle does not directly represent the actual infiltration behavior of the gel electrolyte, the electrochemical performance discussed below was analyzed by considering the Raman characteristics, sheet resistance, and electrode morphology in addition to the wettability. The material and morphological characteristics of the electrodes for each defocusing condition are summarized in Table 2.

### 3.5. Electrochemical Performance of the MSCs

**Figure 7:**
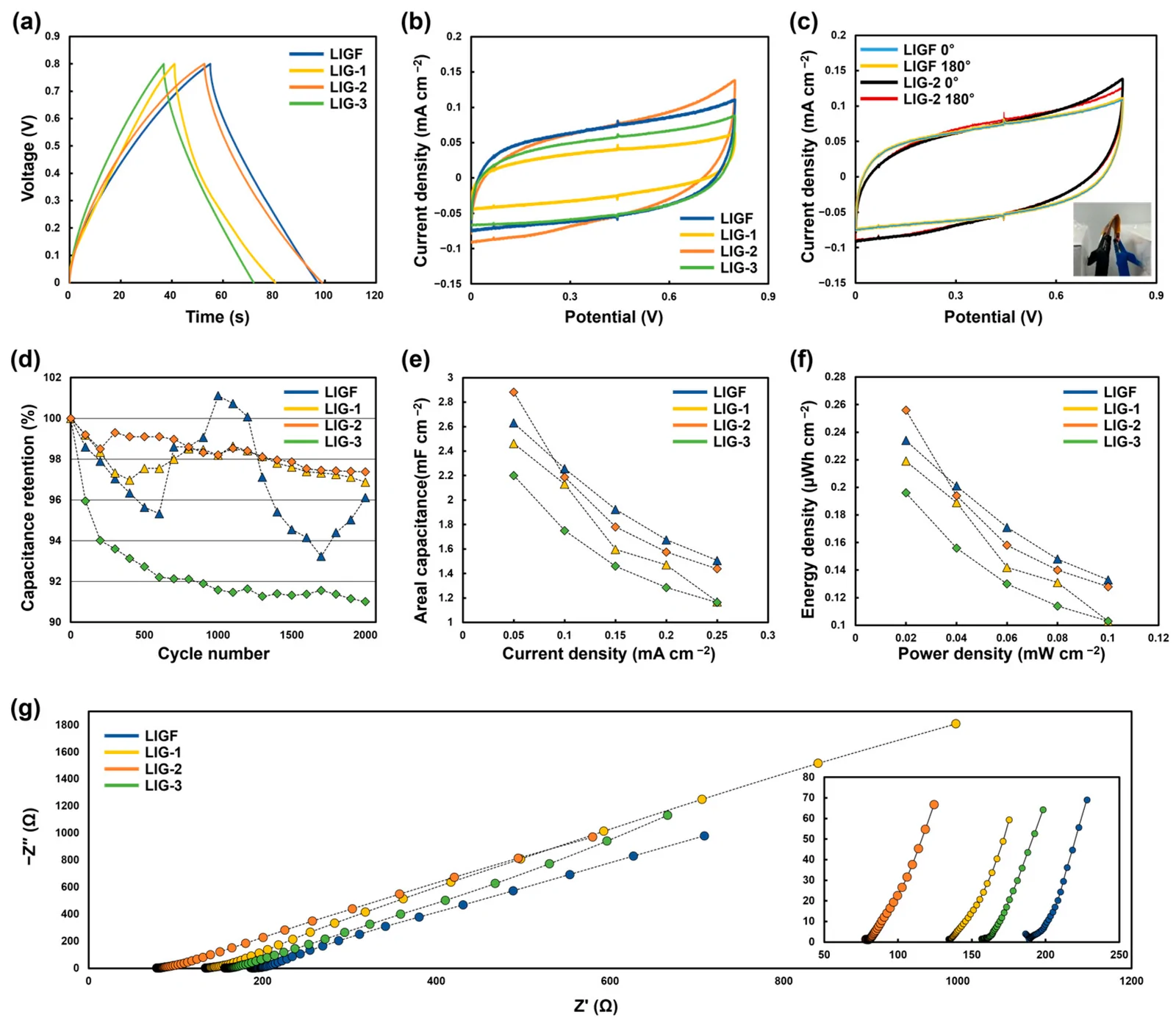
Electrochemical characteristics of the MSCs fabricated under the different defocusing conditions. (**a**) GCD curves at 0.05 mA cm^−2^. (**b**) CV curves at 10 mV s^−1^. (**c**) CV curves of the LIGF and LIG-2 devices in the flat (0°) and 180° bent states (inset: photograph of a device in the bent state). (**d**) Cycling stability over 2000 cycles measured at 50 mV s^−1^. (**e**) Areal capacitance as a function of current density (0.05–0.25 mA cm^−2^). (**f**) Ragone plot. (**g**) EIS Nyquist plots (100 kHz–0.1 Hz; inset: magnified view of the high-frequency region).

Figure 7 summarizes the electrochemical characteristics of the MSCs fabricated under the different defocusing conditions. The GCD curves recorded at 0.05 mA cm^−2^ (Figure 7a) exhibit quasi-triangular profiles without distinct redox plateaus, indicating predominantly electric double-layer capacitor (EDLC) behavior [18]. The discharge times follow the order LIG-2 (46.1 s) > LIGF (42.1 s) > LIG-1 (39.4 s) > LIG-3 (35.2 s), indicating that the stored charge follows the same order. An IR drop corresponding to no more than 10% of the voltage window is observed at the onset of discharge for all electrodes.

The Coulombic efficiencies, calculated as the ratio of the discharge time to the charge time from the GCD curves, are 95.8% for LIG-1, 95.5% for LIG-3, 87.3% for LIG-2, and 76.4% for LIGF. Despite its high stored charge, LIGF exhibits the lowest Coulombic efficiency, indicating relatively large irreversible losses during charging and discharging. However, because the leakage current and self-discharge were not measured separately, the mechanism responsible for these losses could not be identified.

The CV curves recorded at 10 mV s^−1^ (Figure 7b) exhibit quasi-rectangular profiles without distinct redox peaks, further supporting predominantly EDLC behavior. The integrated CV areas follow the order LIG-2 > LIGF > LIG-3 > LIG-1. The two largest CV areas are observed for LIG-2 and LIGF, consistent with the GCD results.

To compare the electrochemical responses under a single bending condition, the CV curves of LIGF and LIG-2 were recorded while the devices were bent to 180° and compared with the corresponding curves obtained in the flat state (0°) (Figure 7c). The retention values calculated from the integrated CV areas were approximately 100% for both electrodes (99.8% for LIGF and 100.8% for LIG-2), indicating negligible changes in the CV response under a single 180° bend relative to the flat state. These results represent the retention of the electrochemical response under a single bending condition rather than durability under repeated bending [18]. Because practical wearable applications require electrode properties to be verified over repeated bending–unbending cycles on the order of 1000 or more, cyclic bending durability testing under such conditions is identified as a necessary subject of future work.

In the cycling stability tests conducted by CV cycling at 50 mV s^−1^ (Figure 7d), the capacitance retention values after 2000 cycles are 97.4% for LIG-2, 96.9% for LIG-1, 96.1% for LIGF, and 91.0% for LIG-3. All four electrodes retain at least 91.0% of their initial capacitance after 2000 CV cycles. Notably, LIG-2 combines the highest initial areal capacitance with the highest capacitance retention of 97.4%.

Figure 7e shows the variation in areal capacitance with current density. At 0.05 mA cm^−2^, the areal capacitances are 2.88 mF cm^−2^ for LIG-2, 2.63 mF cm^−2^ for LIGF, 2.46 mF cm^−2^ for LIG-1, and 2.20 mF cm^−2^ for LIG-3, with LIG-2 exhibiting the highest value. At current densities of 0.10 mA cm^−2^ or higher, however, LIGF surpasses LIG-2, reversing their performance ranking. At 0.25 mA cm^−2^, the areal capacitances are 1.51 mF cm^−2^ for LIGF, 1.44 mF cm^−2^ for LIG-2, 1.17 mF cm^−2^ for LIG-1, and 1.16 mF cm^−2^ for LIG-3. The capacitance retention values at 0.25 mA cm^−2^ relative to those at 0.05 mA cm^−2^ are 57.3% for LIGF, 52.9% for LIG-3, 49.9% for LIG-2, and 47.5% for LIG-1, indicating that LIGF exhibits the best rate capability.

At 0.05 mA cm^−2^, LIG-2 exhibited the highest areal capacitance, consistent with its low sheet resistance and I_D_/I_G_ ratio. In contrast, LIG-3 exhibited the lowest capacitance, consistent with the restricted access of the electrolyte through its surface skin (Section 3.3). At current densities of 0.10 mA cm^−2^ or higher, LIGF surpassed LIG-2 and showed the highest capacitance retention. Although LIGF exhibited the largest equivalent series resistance (ESR), its fibrous morphology and low water contact angle may be associated with improved electrolyte accessibility. However, the static water contact angle does not directly quantify the infiltration behavior of the PVA/H_2_SO_4_ gel electrolyte. Therefore, the observed rate capability should be interpreted by considering the electrode morphology, Raman characteristics, electrical conductivity, and electrolyte accessibility collectively.

The areal capacitance values reported in this study were calculated using the estimated carbonized areas derived from the measured finger widths: 0.862 cm^2^ for LIGF, 0.896 cm^2^ for LIG-1, 0.930 cm^2^ for LIG-2, and 0.918 cm^2^ for LIG-3. Because the measured finger width varied with defocusing, normalization based on the common designed area relatively favored electrodes with wider heat-affected zones. Recalculation based on the designed active area of 0.672 cm^2^ yielded areal capacitances of 3.38, 3.28, 3.99, and 3.01 mF cm^−2^ for LIGF, LIG-1, LIG-2, and LIG-3, respectively. The performance ranking at 0.05 mA cm^−2^ was identical under both normalization bases; however, the difference between LIG-2 and LIGF decreased from 18.0% on the designed-area basis to 9.5% on the estimated-carbonized-area basis. These results show that the area-normalization basis affects the magnitude of the performance difference. When considered together with its lower sheet resistance and I_D_/I_G_ ratio, higher Coulombic efficiency than LIGF, and higher cycling retention, LIG-2 showed a relative advantage at 0.05 mA cm^−2^.

The Ragone plot in Figure 7f mirrors the trend observed for the areal capacitance. At the lowest power density of 0.020 mW cm^−2^, LIG-2 exhibits the highest areal energy density of 0.256 µWh cm^−2^, whereas LIGF, LIG-1, and LIG-3 exhibit values of 0.234, 0.219, and 0.196 µWh cm^−2^, respectively. At a power density of 0.100 mW cm^−2^, however, LIGF exhibits the highest areal energy density of 0.134 µWh cm^−2^, followed by LIG-2 (0.128 µWh cm^−2^), LIG-1 (0.104 µWh cm^−2^), and LIG-3 (0.103 µWh cm^−2^). Thus, LIG-2 is relatively advantageous in the low-power regime, whereas LIGF is more advantageous in the high-power regime.

EIS measurements were performed to investigate the resistive components associated with the observed differences in rate capability and energy–power characteristics. The Nyquist plots in Figure 7g show no distinct high-frequency semicircle within the measured frequency range, while −*Z*″ increases with decreasing frequency. The ESR values estimated from the intercepts with the real axis are 78 Ω for LIG-2, 134 Ω for LIG-1, 157 Ω for LIG-3, and 186 Ω for LIGF [19]. The lowest ESR observed for LIG-2 is consistent with its lowest sheet resistance. In contrast, LIGF exhibits the largest ESR despite its intermediate sheet resistance. This behavior may be attributed to limited inter-fiber contact within the fibrous structure of LIGF, resulting in insufficiently interconnected in-plane conductive pathways. However, because the ESR includes the electrode resistance, current-collector contact resistance, and electrolyte ionic resistance, the differences among the electrodes cannot be attributed to any single factor. The geometric changes identified in Section 3.4 should also be considered when interpreting these resistive components. The narrowing of the inter-finger gap from the designed 0.8 mm to 0.34–0.46 mm shortens the ion-transport path between adjacent fingers, and the measured ESR values are consistent with this effect: LIG-2, with the narrowest gap (0.34 mm), exhibited the lowest ESR, whereas LIGF, with the widest gap (0.46 mm), exhibited the highest. The rate capability followed the opposite order, with LIGF retaining 57.3% of its capacitance at 0.25 mA cm^−2^ compared with 49.9% for LIG-2, indicating that, within the measured gap range, the rate behavior is governed primarily by the electrode morphology rather than by the inter-finger spacing. No evidence of short-circuiting associated with the reduced gap was observed: the CV curves retained quasi-rectangular profiles, and the capacitance retention after 2000 cycles was 91.0–97.4% for all devices. In addition, the lowest Coulombic efficiency (76.4%) was measured for LIGF, which had the widest gap, indicating that the efficiency loss is not associated with gap narrowing.

**Figure 8:**
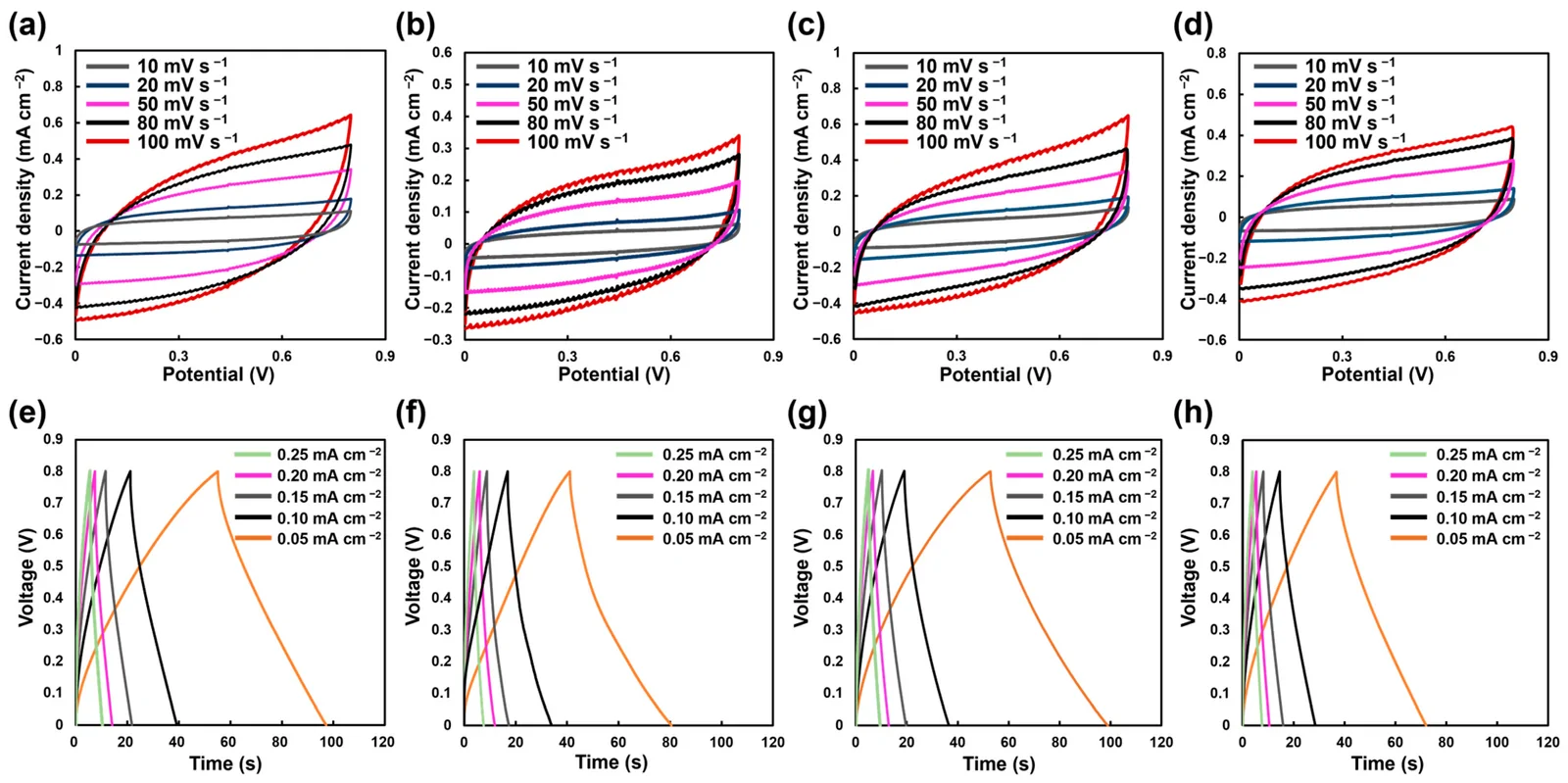
CV curves at different scan rates (10–100 mV s^−1^) for (**a**) LIGF, (**b**) LIG-1, (**c**) LIG-2, and (**d**) LIG-3, and GCD curves at different current densities (0.05–0.25 mA cm^−2^) for (**e**) LIGF, (**f**) LIG-1, (**g**) LIG-2, and (**h**) LIG-3.

The CV curves at different scan rates and the GCD curves at different current densities for each electrode are shown in Figure 8. The CV curves remained quasi-rectangular up to 50 mV s^−1^; at 80 and 100 mV s^−1^, their corners became progressively rounded and the profiles increasingly tilted, although no redox peaks emerged at any scan rate for any of the electrodes. The GCD curves retained their quasi-triangular profiles over the current-density range of 0.05–0.25 mA cm^−2^. Thus, all four electrodes exhibited predominantly EDLC-type behavior over the measured ranges. The distortion observed at high scan rates is consistent with an increasing influence of series resistance rather than a change in the dominant charge-storage mechanism. Consistent with this interpretation, the IR drop at the onset of discharge increased from approximately 2.3–2.4% of the voltage window at 0.05 mA cm^−2^ to 10.6–11.4% at 0.25 mA cm^−2^. Overall, the defocusing distance is a key processing variable that affects MSC performance by altering the electrode morphology and the Raman and electrical characteristics. The main electrochemical performance indicators are summarized in Table 3.

To place these values in context, Table 4 compares the present devices with reported LIG MSCs employing gel polymer electrolytes and no pseudocapacitive modification. Two factors dominate the spread of the reported areal capacitances. The first is the interdigitated geometry. The areal capacitance of LIG MSCs has been shown to increase as the inter-finger spacing decreases, owing to the shortened ion migration distance [25]. The devices reporting the highest values employ finger widths of 0.6–1.0 mm with gaps of 0.3–0.45 mm [18,26] or a comparable finger pitch of 1.3 mm [17]. In the present devices, the measured gap of 0.34–0.46 mm falls within this range, but the carbonized finger width of 1.54–1.66 mm is approximately twice as large and the finger pitch of 2.0 mm exceeds the 1.05–1.3 mm of those devices, so that a greater fraction of the electrode volume lies distant from the electrolyte-facing edge. The second factor is the measurement configuration: for the same unmodified electrode, a three-electrode measurement in a liquid electrolyte has been reported to yield an areal capacitance approximately 20 times that of the corresponding two-electrode device with a gel electrolyte [17]. When the comparison is restricted to two-electrode devices with PVA/H_2_SO_4_ gel electrolyte and millimeter-scale geometries, the present values of 2.20–2.88 mF cm^−2^ lie within the reported range, and the ESR of LIG-2 (78 Ω) is lower than the 176–263 Ω and 194 Ω reported for comparable unmodified devices [17,25]. The coarse interdigitated layout adopted here was necessary to resolve the defocusing-induced broadening of the carbonized fingers described in Section 3.4, and the present study is therefore directed at the systematic mapping of a process parameter rather than at maximizing the areal capacitance.

### 3.6. Performance Trade-Off

In this study, LIGF exhibited the lowest water contact angle and the highest rate capability, whereas LIG-2 exhibited the lowest sheet resistance and I_D_/I_G_ ratio, the highest areal capacitance at 0.05 mA cm^−2^, and the highest cycling retention. The difference between LIG-2 and LIGF was 9.5% on the estimated-carbonized-area basis, and LIGF surpassed LIG-2 at current densities of 0.10 mA cm^−2^ or higher. Therefore, the relative performance of LIG-2 and LIGF depended on the applied current density. Within the measured range, LIG-2 exhibited the highest energy density at 0.020 mW cm^−2^, whereas LIGF exhibited the highest energy density at 0.100 mW cm^−2^. These results indicate that the defocusing distance can be used to tune the electrode morphology and electrochemical performance according to the intended operating condition.

## 4. Conclusions

In this study, PI-based LIG/LIGF electrodes were fabricated by adjusting the defocusing distance of a CO_2_ laser from 0 to 3 mm, and the effects of defocusing on the electrode morphology, Raman characteristics, sheet resistance, patterning fidelity, surface wettability, and MSC performance were investigated. Baseline conditions of 5.0 W and 46 mm s^−1^ were selected by evaluating the laser power and scan speed. As the defocusing distance increased, the surface morphology changed from a fibrous structure to porous LIG, an open-cell-like pore network, and finally a smooth structure with few pores. Within the present test range, LIG-2 exhibited the lowest I_D_/I_G_ ratio (0.29) and sheet resistance (15.3 Ω sq^−1^). Based on the estimated carbonized area, LIG-2 exhibited an areal capacitance of 2.88 mF cm^−2^ and an areal energy density of 0.256 µWh cm^−2^ at 0.05 mA cm^−2^, together with a capacitance retention of 97.4% after 2000 cycles.

Increasing the defocusing distance from 0 to 2 mm decreased both the sheet resistance and I_D_/I_G_ ratio but increased the water contact angle from 12.1° to 75.9°, indicating reduced water wettability. LIGF exhibited the lowest water contact angle and the highest rate capability, whereas LIG-2 showed relative advantages in sheet resistance, I_D_/I_G_ ratio, areal capacitance at 0.05 mA cm^−2^, and cycling retention. The difference in areal capacitance between LIG-2 and LIGF was 18.0% on the designed-area basis and 9.5% on the estimated-carbonized-area basis, demonstrating that the area-normalization basis affects the magnitude of the performance difference. Therefore, MSC performance should be assessed by considering the electrode morphology, Raman characteristics, electrical conductivity, Coulombic efficiency, rate capability, and cycling retention collectively rather than by relying on a single indicator.

Under a single 180° bend, LIGF and LIG-2 retained 99.8% and 100.8% of their flat-state integrated CV areas, respectively, indicating negligible changes in their CV responses. This measurement characterizes the instantaneous response under deformation, and cyclic bending durability over 1000 or more cycles remains to be evaluated. The measured finger widths were 28.3–38.3% greater than the designed value under the tested conditions, while defocusing from 0 to 2 mm increased the finger width by approximately 7.8% relative to the focal condition. Pre-compensation of the electrode dimensions is therefore required to maintain patterning accuracy under different defocusing conditions. Overall, these results demonstrate that defocusing is a simple process-control parameter for tuning the morphology, electrical characteristics, and electrochemical performance of LIG-based MSCs.

## Figures and Tables

**Table 1:** Beam parameters and peak fluence for each defocusing condition, obtained by fitting Equation (1) to the measured carbonized line widths. The effective spot diameter includes the contribution of lateral heat diffusion. The number of overlapping pulses is the ratio of the effective spot diameter to the pulse pitch (25.4 µm at 1000 PPI) along the scan direction. Line widths are given as the mean ± standard deviation of five measurements.

Sample	Defocusing Distance (mm)	Measured Line Width (µm)	Effective Spot Diameter (µm)	Peak Fluence per Pulse (J cm^−2^)	Overlapping Pulses
LIGF	0	227.7 ± 5.4	205	16.7	8.1
LIG-1	1	266.4 ± 5.6	274	9.4	10.8
LIG-2	2	302.0 ± 5.1	417	4.0	16.4
LIG-3	3	254.6 ± 5.2	582	2.1	22.9

**Table 2:** Material and morphological characteristics of the LIG/LIGF electrodes as functions of the defocusing distance. *R*_s_ denotes the sheet resistance. The deviation is the increase in the measured finger width relative to the designed value (1.2 mm); the carbonized area was calculated from the measured finger width, an effective length of 7 mm, and eight fingers.

Sample	Defocusing Distance (mm)	*R*_s_ (Ω sq^−1^)	I_D_/I_G_	I_2D_/I_G_	Finger Width (mm)	Deviation (%)	Carbonized Area (cm^2^)	Contact Angle (°)
LIGF	0	28.7 ± 1.4	0.98	0.61	1.54	+28.3	0.862	12.1 ± 2.6
LIG-1	1	24.4 ± 1.3	0.40	0.60	1.60	+33.3	0.896	44.3 ± 7.6
LIG-2	2	15.3 ± 0.8	0.29	0.50	1.66	+38.3	0.930	75.9 ± 5.7
LIG-3	3	41.9 ± 3.9	1.13	0.36	1.64	+36.7	0.918	33.3 ± 14.7

**Table 3:** Electrochemical performance of the MSCs for each defocusing condition. *C*_A_ and *E*_A_ were calculated using the estimated carbonized area derived from the measured finger width of each electrode; the designed-area-basis column gives the values converted to the common designed active area (0.672 cm^2^). The rate capability is the capacitance retention at 0.25 mA cm^−2^ relative to 0.05 mA cm^−2^. Δ*t* is the discharge time. ^a^ Measured at 0.05 mA cm^−2^.

Sample	*C*_A_ (mF cm^−2^) ^a^	*C*_A_, Designed-Area Basis (mF cm^−2^) ^a^	Δ*t* (s) ^a^	Coulombic Efficiency (%) ^a^	*E*_A_ (µWh cm^−2^) ^a^	Rate Capability (%)	Retention After 2000 Cycles (%)
LIGF	2.63	3.38	42.1	76.4	0.234	57.3	96.1
LIG-1	2.46	3.28	39.4	95.8	0.219	47.5	96.9
LIG-2	2.88	3.99	46.1	87.3	0.256	49.9	97.4
LIG-3	2.20	3.01	35.2	95.5	0.196	52.9	91.0

**Table 4:** Comparison of areal capacitance for unmodified LIG-based microsupercapacitors with gel polymer electrolytes. All entries are LIG formed on polyimide without pseudocapacitive modification; the LIG of ref. [17] was transferred onto a self-healing polymer substrate before device assembly, and the ESR range for ref. [25] covers all finger widths and spacings studied. The capacitance value for ref. [25] was estimated from Figure 2g of that publication. For ref. [17], only the finger pitch (finger width plus gap, 1.3 mm; Figure S15b in the Supporting Information of ref. [17]) is specified, and the individual finger width and gap are not reported. For the present work, the measured carbonized finger width and gap are given (designed values: 1.2 and 0.8 mm; Section 3.4). Three-electrode values and CV-derived capacitances are not directly comparable to two-electrode GCD values. n.r. = not reported.

Ref.	Electrolyte/Cell	Finger/Gap (mm)	Measurement Condition	*C*_A_ (mF cm^−2^)	ESR (Ω)
[25]	PVA/H_2_SO_4_, two-electrode	4/0.5	0.01 mA cm^−2^ (GCD)	2.1	176–263
[17]	PVA/H_2_SO_4_, two-electrode	Pitch 1.3	20 mV s^−1^ (CV)	4.9	194
[17]	PVA/H_2_SO_4_, three-electrode	Pitch 1.3	1.0 mA cm^−2^ (GCD)	96.4	n.r.
[18]	PVA/H_2_SO_4_, two-electrode	1/0.3	0.02 mA cm^−2^ (GCD)	>9	n.r.
[26]	PVA/H_2_SO_4_, two-electrode	0.6/0.45	0.05 mA cm^−2^ (GCD)	22.2	n.r.
This work	PVA/H_2_SO_4_, two-electrode	1.54–1.66/0.34–0.46	0.05 mA cm^−2^ (GCD)	2.20–2.88	78–186

## Data Availability

The original contributions presented in this study are included in the article. Further inquiries can be directed to the corresponding authors.
